# Evaluation of Bioactivity of Essential Oils: Cytotoxic/Genotoxic Effects on Colorectal Cancer Cell Lines, Antibacterial Activity, and Survival of Lactic Acid Bacteria

**DOI:** 10.3390/molecules30040890

**Published:** 2025-02-14

**Authors:** Katarína Kozics, Monika Mesárošová, Monika Šramková, Mária Bučková, Andrea Puškárová, Dominika Galová, Domenico Pangallo

**Affiliations:** 1 Cancer Research Institute, Biomedical Research Center, Slovak Academy of Sciences, Dúbravská Cesta 9, 845 05 Bratislava, Slovakia; katarina.kozics@savba.sk (K.K.); monika.mesarosova@savba.sk (M.M.); monika.sramkova@savba.sk (M.Š.); 2 Institute of Molecular Biology, Slovak Academy of Sciences, Dúbravská Cesta 21, 845 51 Bratislava, Slovakia; maria.buckova@savba.sk (M.B.); andrea.puskarova@savba.sk (A.P.); dominika.galova@savba.sk (D.G.)

**Keywords:** essential oils, cytotoxicity, genotoxicity, reactive oxygen species, antibacterial effect, colorectal cancer

## Abstract

Colorectal cancer (CRC) ranks among the most frequently diagnosed malignancies and is associated with a significantly high mortality rate. In recent years, increasing attention has been directed toward naturally derived substances with anticancer properties. In our study, we focused on determining the biological and antibacterial effects of selected essential oils (EOs)—peppermint, oregano, tea tree, lemon, lavender, frankincense, and oil blends (Zengest and OnGuard). Analyses were performed on human colon carcinoma cell lines (HCT-116 and HT-29). The cytotoxic (MTT assay), genotoxic effects (comet assay), and reactive oxygen species levels (ROS-Glo™ H_2_O_2_ Assay) of EOs and oil blends were determined. In our study, we found that all of the studied oils have the potential cyto/genotoxic effects on CRC cell lines after 24 h exposure. The results revealed that oregano, Zengest, and frankincense showed statistically the highest cytotoxic effects [IC_50_ 0.05 µg/mL] compared to the other studied oils. These oils induced DNA damage and also increased ROS levels. On the other hand, peppermint was shown to have the lowest cytotoxic effect [IC_50_ 0.67 µg/mL] on the HT-29 cell line. We also evaluated the antibacterial effects of oregano, tea tree, and the OnGuard blend, determining their impact on the viability of beneficial bacteria models, including *Lacticaseibacillus rhamnosus*, *Lactiplantibacillus plantarum*, *Lacticaseibacillus paracasei*, *Lactobacillus brevis*, *Lactobacillus pentosus*, and *Weizmannia coagulans*. Oregano exhibited strong antibacterial activity, with an inhibition zone of 31 mm, while tea tree and OnGuard showed inhibition zones ranging from 12 to 15 mm. The EOs (oregano, tea tree, OnGuard) demonstrated antibacterial effects, with MICs ranging from 0.05 to 0.5 µg/mL. Peppermint, lemon, lavender, frankincense, and the Zengest blend did not inhibit the growth of lactic acid bacteria or *W. coagulans*, and thus did not impact bacterial survival. On the other hand, they demonstrated potential anticancer effects.

## 1. Introduction

Colorectal cancer (CRC) is the third most common cancer in men and the second in women, with the most common incidence recorded in Central Europe, Australia, and North America. Despite continuous efforts in the field of CRC treatment, the mortality rate remains alarming, as CRC accounted for more than 10% of all cancer deaths in 2020 [1,2]. However, an adequately effective method for CRC treatment and prevention has not yet been established. Currently, treatment typically involves invasive procedures, which may be supplemented with chemotherapy and radiotherapy, either alone or in combination, as adjuvant or neoadjuvant therapies. Additionally, targeted therapy and immunotherapy have emerged as recent therapeutic approaches for managing aggressive, advanced, or metastatic CRC [3,4]. Nonetheless, these strategies show numerous adverse side effects, and as a result, new adjuvant therapeutical tools are being sought.

The study of natural products has long been a cornerstone of applied pharmacology [5]. They have played a key role in drug discovery, particularly for cancer and infectious diseases [6,7]. According to Newman and Cragg [8], natural products continue to provide the greatest potential for identifying new compounds that can develop into effective treatments for a wide range of human diseases.

Essential oils (EOs), which are complex mixtures of volatile organic compounds extracted from plants through steam distillation, dry distillation, or mechanical processes that do not involve heating, exhibit a range of biological and pharmaceutical properties, including anticancer activity [9]. Hence, the use of EOs in the treatment of neoplasia is an increasingly promising area of research [10]. EOs have demonstrated a wide array of bioactive effects, such as cytotoxicity, antiproliferative, and antimetastatic actions on cancer cells, through various mechanisms of action [11]. EO compounds have been found to exert anticancer activity against numerous human neoplastic cell lines, including CRC, either alone or in association with anticancer drugs [12]. For example, Ziziphora EO, which contains anticancer compounds such as menthol and pulegone, caused apoptosis in HT-29 cells in vitro by increasing C3 and C9 expressions and decreasing Bcl-2 expression [13]. Cinnamaldehyde and eugenol, after 72 h of treatment, were also capable of inducing apoptosis, necrosis, and a cell cycle slowdown in CRC cell lines Caco-2 and SW-620 but not in a normal human mucosal epithelial cell line NCM-460 [14]. There are several studies [15,16,17,18,19] examining the antibacterial properties of EOs. However, there is not nearly enough information about the potential risk of using EOs on the beneficial bacteria in the gut. The gut microbiota plays a crucial role in regulating the health and physiology of the host [20]. One of the most popular, widely utilized probiotics for many years until now is the Lactobacillus species, which is the largest heterogeneous group among the lactic acid bacteria (LAB) that do not cause illness. Bacterial species such as *Lactobacillus acidophilus*, *Lactobacillus casei*, *Lactobacillus rhamnosus*, *Lactobacillus plantarum*, *Lactobacillus fermentum*, *Lactobacillus helveticus*, and *Lactobacillus bulgaricus* have beneficial effects on health [21].

In our study, we evaluated the effect of six EOs (peppermint, oregano, tea tree, lemon, lavender, frankincense) and two EO blends (Zengest and OnGuard) on CRC cell lines HT-29 and HCT-116. We determined the cytotoxic and genotoxic effects, as well as reactive oxygen species (ROS) generation. In addition, the studied EOs and EO blends were also screened for antibacterial properties against LAB strains, and changes in the growth and survival of these species were monitored.

## 2. Results

### 2.1. Cytotoxic Effect of EOs and EO Blends

**Figure 1:**
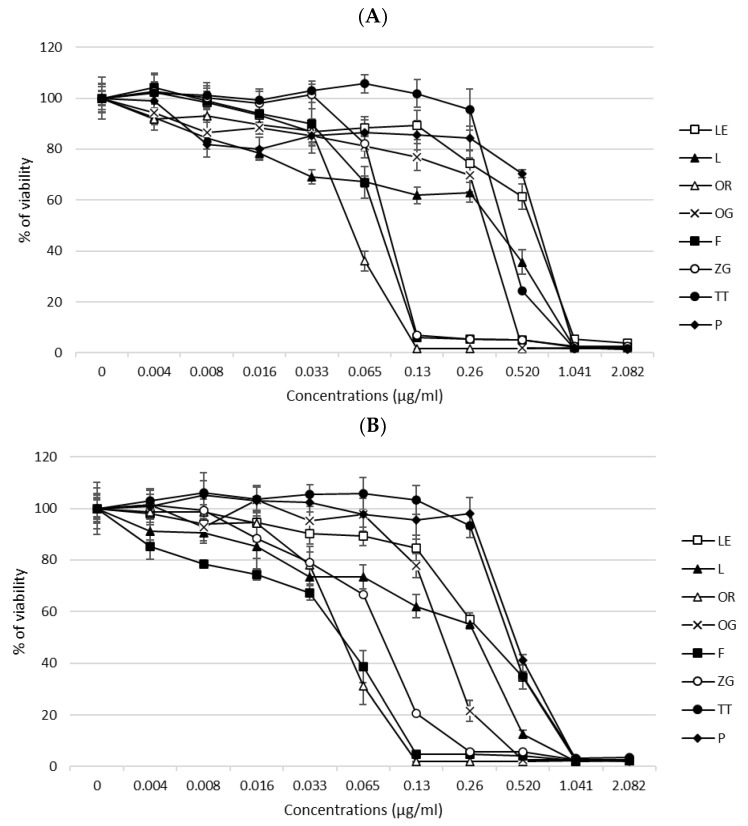
Cytotoxic effects of EOs and EO blends on the CRC cell lines HT-29 (**A**) and HCT-116 (**B**) after 24 h of treatment. Data are represented as means ± SD of three independent experiments.

The cytotoxic effects of EOs (peppermint—P, oregano—OR, tea tree—TT, lemon—LE, lavender—L, frankincense—F) and EO blends (Zengest—ZG and OnGuard—OG) on HT-29 and HCT-116 cells were determined using the MTT assay. Cells were treated with the selected oils for 24 h, and changes in cell viability were noted. The results are presented in Figure 1A,B. After 24 h, a decrease in cell viability was observed in direct dependence on the applied concentration of the compound. IC_50_ values (median inhibitory concentrations that cause approximately 50% cell death) were determined for both cell lines tested (Table 1).

Based on these results, we further focused on determining the genotoxic effects of the selected oils. For the comet assay, we used concentrations of EOs related to the IC_50_ value. In case no DNA damage was detected, higher concentrations were used to prove the cytotoxic as well as genotoxic effects of the EOs.

### 2.2. DNA-Damaging Effects of EOs and EO Blends

The level of DNA strand breaks induced in HT-29 and HCT-116 cells by EOs and EO blends was determined by the comet assay and expressed as % tail DNA. All studied EOs induced DNA damage compared to untreated control cells (Table 2A,B). All selected EOs (except ZG) significantly increased DNA damage, with higher viability of HT-29 and HCT-116 cells at IC_30–50_. The most prominent effects were observed with LE, P, and OG, which demonstrated genotoxicity at ~80% viability for HT-29 cells. OR proved to have the highest genotoxicity at a concentration of 0.033 µg/mL in the HT-29 cell line.

### 2.3. ROS Production

**Figure 2:**
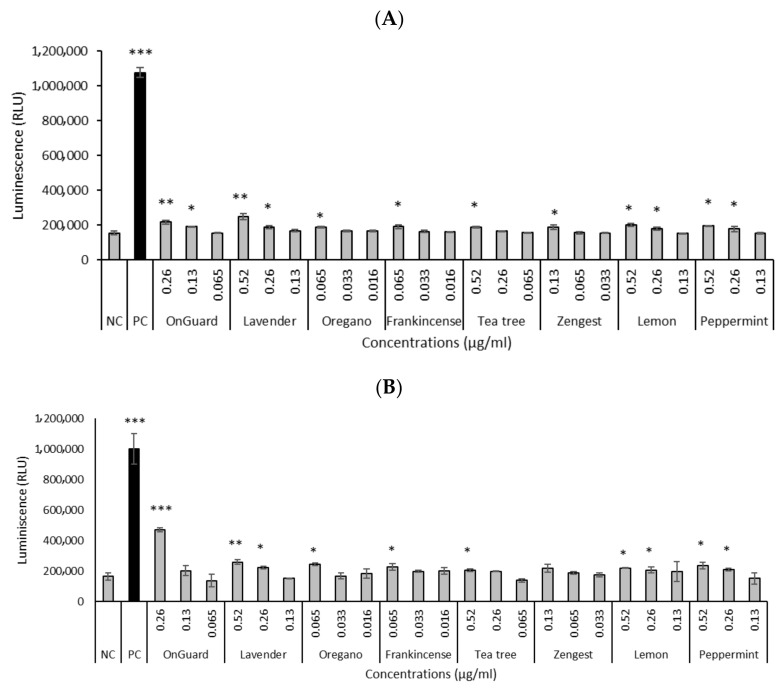
Effect of EOs and EO blends on ROS production after 24 h in the HT-29 (**A**) and HCT-116 (**B**) cell lines. Positive control (PC)—menadione (50 μmol/L). NC—negative control. Data are presented as means ± SD of three independent experiments. * *p* < 0.05; ** *p* < 0.01; *** *p* < 0.001 indicate statistically significant differences compared to the negative control (Student’s *t*-test).

Based on the genotoxicity results, we further focused on determining the production of ROS of the selected oils. ROS production was analyzed using the ROS-Glo™ H_2_O_2_ Assay. Selected oils, at concentrations that increased DNA damage, significantly increased ROS production in both studied cell lines (Figure 2). All EOs significantly increased ROS production at higher concentrations for both cell lines. The significantly highest ROS production was shown by OG at a concentration of 0.26 µg/mL for HCT-116 cells (Figure 2B).

### 2.4. Determination of the Antibacterial Activity of EOs

**Figure 3:**
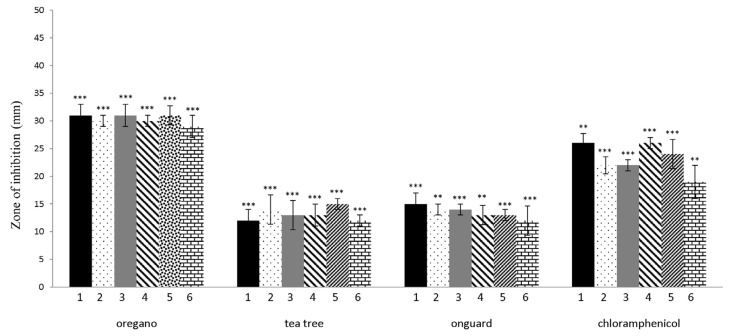
Effect of EOs and OG blend on LAB and W. coagulans, determined by the disc method. Strains were incubated for approximately 24 h at 37 °C. Chloramphenicol (30 μg/disc) was used as a positive control. Each bar of the chart represents the mean of the inhibitory zones obtained for EOs analyzed in (1) *L. plantarum*, (2) *L. rhamnosus*, (3) *L. pentosus*, (4) *L. paracasei*, (5) *L. brevis*, and (6) *W. coagulans*. Data are presented as means ± SD of three independent experiments. ** *p* < 0.01; *** *p* < 0.001 indicate statistically significant differences compared to the control (Student’s *t*-test).

The in vitro antibacterial activity of six EOs and two EO blends against LAB and *W. coagulans* was assayed using the disc diffusion method by measuring inhibition zone diameters (Figure 3). OR, TT, and OG showed antibacterial effects based on these inhibition zones (* *p* < 0.05; ** *p* < 0.01; *** *p* < 0.001). OR was extremely effective on all tested bacteria, with inhibition zones ranging from 29 to 31 mm. The differences in the measured inhibition halos of OR (*p* = 0.000456), TT (*p* = 0.000459), and OG (*p* = 0.0119) on *L. rhamnosus* and *L. paracasei* were statistically different from the negative control (without the use of EOs). The inhibition halo produced by OR was much larger than those of chloramphenicol in all tested isolates. All tested isolates were sensitive to the EO of TT and the EO blend OG (mean inhibition diameter ranging from 12 to 15 mm). The inhibition zones of the tested TT and OG blend were significantly lower than the positive controls, represented by chloramphenicol (19–26 ± 2.5 mm). EOs LE, P, L, F, and EO blend ZG did not show antibacterial activity (no inhibition zones) against the LAB, *W. coagulans*, and therefore, were not tested in the broth dilution method.

### 2.5. Evaluation of Minimum Inhibitory Concentration (MIC) in Liquid Medium

The screening revealed that the EOs OR, TT, and the OG blend had an inhibitory effect against all tested bacteria; therefore, an additional MIC assay was performed with these three EOs. The results obtained from the MIC assay are shown in Table 3. These antibacterial assays revealed that OR exhibited very strong activity (MIC 0.05 µg/mL), while TT and the OG blend had less antibacterial activity (MIC 0.5 µg/mL). All three EOs inhibited the growth of bacteria (*L. rhamnosus*, *L. plantarum*, *L. paracasei*, *L. brevis*, *L. pentosus*, and *W. coagulans*) by evaluation of MIC.

### 2.6. Determination of the Number of Viable Bacteria

Four EOs (LE, P, L, F) and the ZG blend, which did not show antibacterial effects, were screened at a concentration of 1.25 µg/mL on the survival of LAB and *W. coagulans*. The results are summarized in Table 4. All tested strains were able to tolerate and grow in a liquid medium supplemented with EOs. It was found that the levels of bacteria *L. plantarum* and *L. rhamnosus* increased in the medium supplemented with LE, P, and L by 1 log CFU/mL order compared to the control (without the EOs). F and ZG blend did not show an increase in bacterial growth in all tested strains.

## 3. Discussion

CRC has become a great threat in most developed countries over recent decades. A large body of evidence shows that lifestyle and dietary habits, in particular, play some predominant role in the onset of many malignancies, including CRC [22]. On the other hand, cancer prevention using food phytochemicals obtained from biologically active plants is an attractive and reasonable strategy, especially when considering traditional uses in local remedies as well as the unique dietary habits of indigenous populations [23]. Indeed, the active constituents of spices and herbs have been shown to have marked potential for cancer prevention [24]. Many scientific studies have confirmed the therapeutic effect of EOs on various types of cancer cells [25,26,27]. In our study, we aimed to determine the effect of EOs and EO blends on the CRC tumor cell lines, as the incidence of this disease is steadily increasing.

Effects such as cytotoxicity, genotoxicity, ROS production, and the antibacterial effects of EOs and EO blends were determined in this study. The cytotoxic effects of the selected oils on CRC cells were evaluated using the MTT assay, and the results showed that the 24 h-treatment with EOs and EO blends affected cell viability in a dose-dependent manner. Selected compounds exhibited variable potencies (IC_50_) according to the following sequence: ZG < OR < F < OG < L < TT < LE < P for the HT-29 cell line, and ZG = F < OR < OG < LE < L < TT < P for the HCT-116 cell line. All eight compounds showed a cytotoxic effect to some extent. ZG, OR, and F (IC_50_~0.05–0.08 µg/mL) showed the highest cytotoxicity in both cell lines, while the lowest was determined for P (IC_50_~0.67 µg/mL) in the HT-29 cell line. The cytotoxic effects of EOs have been described in several studies. Begnini et al. investigated the effects of the EO *Origanum vulgare* on HT-29 colon and MCF-7 breast tumor cells. Their results showed that the EO is composed mostly of 4-terpineol and induces a high cytotoxic effect in HT-29 cells, while in the MCF-7 cell line, the EO was less effective [28]. Zengin et al. [29] tested the effects of three solvent extracts (ethyl acetate, methanol, and water) from *Origanum sipyleum* on the viability and spontaneous migration of the HCT-116 cell line. The MTT test showed that the methanol and water Origanum extracts (100 μg/mL) could be considered biocompatible, with resulting cell viability of ≥70% and ≤130% compared to vehicle-treated cells. On the contrary, the ethyl acetate extract significantly reduced cell viability (<60% compared to the control group). Fahmy et al. [30] evaluated the anticancer activity of the EO from *Lavandula officinalis* against six human cancer cell lines: hepatocellular carcinoma (HepG2), prostate (PC3), lung carcinoma (A549), skin cancer (A431), colon cancer (HCT-116), and breast cancer (MCF7). The EO had a highly cytotoxic effect on the HepG2 and A549 cell lines, with 100% death at 100 μg/mL and IC50 of 67.8 and 12 μg/mL, respectively, while its activity on the other cell lines (HCT-116, MCF-7) was weaker. In addition, Jayaprakasha et al. [31] found that limonene acid and β-sitosterol glucoside from *Citrus aurantium* L. were effective agents in promoting apoptosis in human colon cancer cells (HT-29) as well as non-cancerous cells (COS-1). These compounds did not show any toxic effects on non-cancerous cells but caused a 4–5-fold increase in the number of HT-29 cells in the G2/M phase at 50 μM, suggesting a potential role in cell cycle arrest. These findings support the hypothesis that limonoids and phytosterols, incorporated in enriched fractions of these compounds into the diet, may serve to prevent colon cancer.

We also focused on the genotoxic effect, determined by the comet assay. For all selected oils, we observed a significant genotoxic effect in both cell lines. The most prominent were OG, P, and LE, which demonstrated genotoxicity at concentrations causing ~80% viability. OR proved to have the highest genotoxicity at a concentration of 0.033 µg/mL on the HT-29 cell line. In addition, F, at a concentration of 0.065 µg/mL, also showed a statistically significant increase in DNA damage. At non-cytotoxic doses (>IC_80_), EOs are generally not genotoxic in the comet assay and are mainly characterized by protective activity [32]. On the other hand, some EOs, or their components, induce DNA damage even if they are not cytotoxic. For example, derivatives of thymol showed significantly increased DNA damage in HT-29 and HCT-116 cell lines at a non-cytotoxic concentration [33]. EOs extracted from *L. angustifolia* and *Q. infectoria* by increasing oxidative/nitrosative stress (decreasing SOD, GSH-Px, and CAT, and increasing MDA, PCO, and NO) and DNA damage may be effective in treating *M. marshalli* infections [34].

ROS are highly reactive molecules produced in cells as a response to external stimuli or stress. Under physiological conditions, ROS levels are kept low and tightly regulated, playing a crucial role in cellular signaling and maintaining homeostasis. Cancer cells exhibit elevated basal levels of ROS, and increased expression of the antioxidant system supports their survival, which is a major cause of drug resistance [35]. Therefore, it has been proposed that agents that can cause the elevation of ROS by disturbing the balance of the inbuilt redox system could serve as a potential and safe drug [36]. This effect has been observed in cancer cells treated with EOs. For example, a study found that EOs from *Aniba rosaeodora* (rosewood) induced apoptosis in human epidermoid carcinoma A431 and immortalized HaCaT cells through ROS generation [37]. Similar effects were seen with EOs from *Zanthoxylum schinifolium* in hepatocarcinoma cells HepG2 [38].

In this study, to test the level of EO-induced ROS generation, we used the ROS-Glo™ H_2_O_2_ Assay. All selected EOs and EO blends produced ROS at cyto/genotoxic concentrations, which could explain the detected DNA damage.

Results of Islam et al. [39] suggest that EOs isolated from *Calocedrus fomosana* (CF) act as a promising anticancer agent against colon cancer cells. In this study, CF significantly induced ROS-mediated autophagy and apoptosis in the HCT-116 cell line. Oxidative stress activation was also observed in thymol-treated B16 melanoma cells and non-small lung cancer (A549) cells. This confirmed that ROS-mediated toxicity is the principal cancer-killing mechanism of thymol [40,41]. Applying redox-active copper (II) with thymoquinone (EOs of the *Nigella sativa* plant seeds) increases DNA damage, apoptosis, and cell death by increasing the amount of intracellular ROS through pro-oxidant activity in the HT-29 cell line [42].

Although the inhibition of pathogenic microorganisms with EOs has been shown [15,16,43,44,45], only relatively few studies [46,47,48] have measured the effects of EOs on LAB.

Our study compared the antibacterial efficacy of six different EOs and EO blends against five models of beneficial bacteria such as *L. rhamnosus*, *L. plantarum*, *L. paracasei*, *L. brevis*, *L. pentosus,* and *W. coagulans*. As indicated by the experimental results, it is now clear that only OR, TT EOs, and the OG blend provided effective inhibition of the growth of the above-mentioned bacterial isolates. The antibacterial analysis showed that EO from *Origanum vulgare* exhibits inhibition against all bacteria tested at a concentration of 0.05 µg/mL. In the case of TT and OG, the MIC concentration was detected at 0.25 or 0.5 µg/mL. Similarly, Horošová et al. [49] reported a strong bactericidal effect with oregano EO against lactobacilli and *E. coli* with a MIC of 0.05%. Moritz et al. [50] obtained MIC values for clove and mint EOs of 0.2 and 0.4% *v*/*v*, respectively, against *L. rhamnosus*. Comparable antibacterial activities of EOs of clove bud, cinnamon bark, and thyme, and their individual compounds were monitored by Dunn et al. [51] against 9 LAB species. The effect of thymol derivatives against lactobacilli strains and *W. coagulans* in the agar-diffusion method, as well as the determination of the number of colonies on the plates, was described by Blažíčková et al. [33]. Disc diffusion is a reliable standard method for assessing the antimicrobial susceptibility of microorganisms [52]. Based on the results of this method, we categorized the tested oils into two groups: one consisting of oils with antibacterial potential (oregano, tea tree, OnGuard) and the other comprising oils that showed no antibacterial effect (peppermint, lemon, lavender, frankincense, and the Zengest blend). The EOs without antibacterial activity were considered to have potential protective properties. Sarabi-Jamab and Niazmand [53] evaluated the effect of the EOs from *Mentha piperita* and *Ziziphora clinopodioides* on the growth of *L. acidophilus* as a biostarter culture. There was no difference in the viability of *L. acidophilus* among samples containing various concentrations of EOs and the control. Our results demonstrate that all tested strains were able to tolerate and grow in a liquid medium supplemented with EOs. The growth of LAB was actually increased by the addition of peppermint, lemon, and lavender.

## 4. Materials and Methods

### 4.1. Essential Oils

Commercial EOs used in this study were as follows: lemon (LE) from *Citrus limon* L., peppermint (P) from *Mentha piperita* L.; lavender (L) from *Lavandula angustifolia* Mill.; frankincense (F) from *Boswellia frereana* Birdw., *Boswellia carterii* Flueck., and *Boswellia sacra* Flueck.; oregano (OR) from *Origanum vulgare* L.; tea tree (TT) from *Melaleuca alternifolia* Cheel.; and oil blends Zengest (ZG) and OnGuard (OG). EOs were purchased from doTERRA (Pleasant Grove, UT, USA) and stored in a dry and dark place. The chromatograms and the peak reports of the six essential oils are described in the Supplementary Materials Section. The EOs were weighed to determine the volume that comprised 10 mg. This amount was used in testing as the full-strength (100%) concentration. The EOs were diluted in dimethyl sulfoxide (DMSO; Merck KGaA, Darmstadt, Germany). During the treatment of cell cultures with EOs/EO blends, the DMSO concentration was below 0.04%.

### 4.2. Cell Culture

Colorectal carcinoma cell lines HCT-116 and HT-29 were obtained from American Type Culture Collection (USA). Cells were cultured in Dulbecco’s Modified Eagle Medium (DMEM) in low glucose (1 g/L) with added 10% fetal bovine serum and 1% penicillin-streptomycin (Thermo Fisher Scientific, Waltham, MA, USA). The cells were placed in an incubator at 5% CO_2_ and 37 °C. Media and chemicals used for cell cultivation were purchased from Gibco BRL (Thermo Fisher Scientific, Paisley, UK).

### 4.3. Bacterial Strains

All bacterial strains used as models of beneficial bacteria, including *Lacticaseibacillus rhamnosus*, *Lactiplantibacillus plantarum*, *Lacticaseibacillus paracasei*, *Lactobacillus brevis*, *Lactobacillus pentosus*, and *Weizmannia coagulans*, were obtained from the collection of microorganisms at the Food Research Institute in Bratislava, Slovakia.

### 4.4. Determination of Cytotoxicity (MTT Assay)

The cytotoxicity of selected compounds was determined by the MTT assay (3-(4,5-dimethyl-thiazolyl)-2,5-diphenyltetrazolium bromide) [54]. Briefly, 1 × 10^6^ cells were seeded in 96-well plates and cultured in a complete DMEM medium. Exponentially growing cells were pre-incubated (at 37 °C in a 5% CO_2_ atmosphere) in the presence of different concentrations of the selected EOs and EO blends (0–2.08 µg/mL) for 24 h. Cells treated with the medium only served as a negative control. At the end of the treatment, the samples were washed with phosphate-buffered saline (PBS), followed by incubation with 1 mg/mL of MTT for 4 h. The MTT solution was then removed, and the formazan crystals were dissolved with DMSO for 40 min. Absorbance at a wavelength of 540 nm was measured using an xMark microplate spectrophotometer (Bio-Rad Laboratories, Inc., Hercules, CA, USA), and background absorbance at 690 nm was subtracted. IC_50_ values for selected EOs and EO blends were calculated using linear regression.

### 4.5. Determination of Genotoxicity (Comet Assay, SCGE)

Genotoxicity was determined by the alkaline comet assay (single-cell gel electrophoresis, SCGE), which allows for the detection of DNA breaks [55]. Cells were seeded in a 6-well plate (3 × 10^4^ cells/well) and treated with EOs/EO blends (0–0.52 µg/mL) for 24 h. Lysis was performed in a cooled solution consisting of 2.5 M NaCl, 100 mM Na_2_EDTA, 10 mM Tri-HCl (pH 10), and 1% Triton X-100 for one hour in the cold. The samples were transferred to an electrophoretic solution (300 mM NaOH, 1 mM Na_2_EDTA, pH > 13) in an electrophoretic apparatus and allowed to unwind for 30 min at 4 °C in the dark. Electrophoresis (19 V, 300 mA) was then performed for 20 min at 4 °C. Samples were neutralized by washing in a neutralization solution for 2 × 10 min (0.4 M Tris-HCl, pH 7.4). After the slides had dried, ethidium bromide (5 g/mL) was applied. The slides were examined with a Zeiss Imager.Z2 fluorescence microscope, with computerized image analysis (Metafer 3.6, MetaSystems GmbH, Altlussheim, Germany). The percentage of DNA in the tail was used as a parameter for the measurement of DNA damage (DNA strand breaks). Five hundred comets were scored per sample in one electrophoresis run.

### 4.6. Determination of ROS Production

Oxidative stress was analyzed using the ROS-Glo™ H_2_O_2_ Assay (Promega, Madison, WI, USA). The ROS-Glo™ H_2_O_2_ Assay is a bioluminescent assay that measures the level of hydrogen peroxide (H_2_O_2_), a reactive oxygen species (ROS), directly in cell culture or in defined enzyme reactions. Briefly, 2 × 10^4^ cells were seeded in 96-well plates and cultured in a complete DMEM medium. Exponentially growing cells were pre-incubated (at 37 °C in a 5% CO_2_ atmosphere) in the presence of different concentrations of the selected EOs and EO blends for 24 h. Cells treated with the medium only served as a negative control, and cells treated with menadione (50 μmol/L) served as a positive control. H_2_O_2_ substrate solution was added for 6 h to generate a luciferin precursor according to the manufacturer’s protocol. The addition of ROS-Glo™ Detection Solution (20 min) converts the precursor to luciferin and provides Ultra-Glo™ Recombinant Luciferase to produce a light signal that is proportional to the level of H_2_O_2_ present in the sample. The relative luminescence was measured using a GloMax^®^ Discover Microplate Reader (Promega, Madison, WI, USA).

### 4.7. Evaluation of Antibacterial Activity

The antibacterial effects of the EOs were analyzed by the disc diffusion test under anaerobic conditions [15]. The effect on five LAB (*L. rhamnosus*, *L. plantarum*, *L. paracasei*, *L. brevis*, *L. pentosus*) and one Gram-positive bacterium (*W. coagulans*) was monitored. A single colony from the bacterial culture plate was seeded into 3 mL of nutrient medium (NM) for *W. coagulans* and De Man, Rogosa, and Sharpe (DRS) for LAB bacteria. The culture was carried out anaerobically at 37 °C for 24 h in Anaerocult (Merck KGaA, Darmstadt, Germany). After 24 h, the cultures were spread on appropriate agar plates using a sterile swab. Sterile filter paper discs (6 mm Ø Whatman No. 1) were pressed onto the surface of the agar plates, and EOs were then pipetted onto the discs. Each EO was tested at 100% strength. Chloramphenicol (30 μg/disc; Merck KGaA, Darmstadt, Germany) was used as a positive control. Plates were incubated for approximately 24 h at 37 °C in Anaerocult, and the diameter of the inhibition zones was measured in mm. The inhibition halos of EOs and EO blends were assessed according to Ponce et al. [56] as follows: extremely sensitive for a diameter larger than 20 mm, very sensitive for a diameter of 15–19 mm, and not sensitive for a diameter less than 8 mm.

### 4.8. Evaluation of MIC in Liquid Medium

The MIC of OR and TT EOs, and the EO blend OG was determined using a broth microdilution method in 96-well strip tubes, according to Puškárová et al. [15]. Bacterial suspensions were adjusted to a final concentration of 10^5^ CFU/mL in DRS medium for Lactobacilli and in NM for *W. coagulans*. One hundred microliters of DRS or NM containing 5% DMSO were distributed into the wells of the microtiter plates. The EOs and the EO blend OG (10 µL) were added to these wells at a range of final concentrations: 0.05, 0.25, 0.5, 1.0, and 2.5 µg/mL. One hundred microliters of bacterial suspension were finally added to each. The plates were incubated at 37 °C for 24 h in Anaerocult (Merck, Millipore, Darmstadt, Germany).

### 4.9. Determination of the Number Viable Bacteria

The effect of the EOs LE, P, L, and F, and the EO blend ZG on the survival of LAB and *W. coagulans* was evaluated by the agar plate method for quantifying viable bacteria [57]. Bacterial suspensions were adjusted to a final concentration of 10^5^ CFU/mL in DRS medium for Lactobacilli and in NM for *W. coagulans*. One thousand microliters of DRS or NM containing 5% DMSO were distributed into the wells of the plates. The EOs and the ZG blend were added to these wells at a concentration of 1.25 µg/mL. One hundred microliters of bacterial suspension were finally added to each well. The plates were incubated at 37 °C for 24 h in Anaerocult (Merck, Millipore). The experiments were replicated three times, and the results were expressed as a log of the number of colonies forming units in 1 mL of sample.

### 4.10. Statistical Analysis

The results represent the mean from 3 to 5 experiments ± standard deviation (SD). The differences between the control and treated samples were tested for statistical significance using Student’s *t*-test (* *p* < 0.05; ** *p* < 0.01; *** *p* < 0.001). Because the antibacterial activity datasets were normally distributed, the independent samples *t*-test was performed to test for significant differences between groups.

## 5. Conclusions

In conclusion, our study points to the potential anticancer effects of the tested EOs and EO blends in the context of CRC, as well as their antibacterial activity. All selected EOs/EO blends showed cyto/genotoxic effects and increased the ROS levels in HT-29 and HCT-116 cells. Oregano, frankincense, and Zengest showed the highest cytotoxicity. In addition, in the determination of antibacterial activity, LAB survival was found in the presence of peppermint, lemon, lavender, frankincense, and Zengest.

Overall, this study suggests that frankincense and Zengest may serve as promising candidates for further research and development in cancer prevention and as adjuncts to conventional therapies. However, more detailed studies are necessary to fully understand their mechanisms of action and clinical potential.

## Figures and Tables

**Table 1:** IC_50_ values (calculated by linear regression) for HT-29 and HCT-116 cells after exposure to EOs and EO blends for 24 h.

EOs/EO Blends	IC_50_ (µg/mL)	
	HT-29	HCT-116
LE	0.62	0.34
L	0.38	0.29
OR	0.056	0.052
OG	0.33	0.29
F	0.083	0.05
ZG	0.05	0.05
TT	0.42	0.45
P	0.67	0.48

Notes: LE: lemon, L: lavender, OR: oregano, OG: OnGuard, F: frankincense, ZG: Zengest. TT: tea tree, P: peppermint.

**Table 2:** The levels of DNA single-strand breaks (% of tail DNA) in HT-29 (**A**) and HCT-116 (**B**) cells after exposure to EOs and EO blends for 24 h. Data are presented as means ± SD of three independent experiments. * *p* < 0.05; ** *p* < 0.01; *** *p* < 0.001 indicate statistically significant differences compared to the negative control (Student’s *t*-test).

(**A**)								
Concentrations of EOs and EO blends (µg/mL)								
	0	8 × 10^−3^	1.7 × 10^−2^	3.3 × 10^−2^	6.5 × 10^−2^	0.13	0.26	0.52
OnGuard	9.11 ± 1.15	8.52 ± 2.03	8.92 ± 2.03	9.02 ± 1.05	10.36 ± 2.23	17.02 ± 1.23 **	ND	ND
Lavender	9.95 ± 2.11	9.08 ± 1.68	10.15 ± 2.47	9.22 ± 1.63	9.72 ± 1.51	10.67 ± 1.12	14.74 ± 1.4 *	31.17 ± 1.76 ***
Oregano	8.17 ± 1.25	8.86 ± 1.08	9.31 ± 0.65	14.12 ± 0.33 *	24.31 ± 1.84 ***	35 ± 2.15 ***	ND	ND
Tea tree	7.52 ± 1.81	8.76 ± 0.84	8.12 ± 0.88	9.01 ± 1.05	8.10 ± 0.75	8.85 ± 2.21	11.96 ± 0.71 *	21.38 ± 2.68 **
Zengest	9.39 ± 1.03	8.22 ± 0.93	8.12 ± 0.88	10.42 ± 2.12	9.90 ± 1.32	37.22 ± 4.58 ***	ND	ND
Lemon	8.25 ± 1.34	9.06 ± 0.79	8.54 ± 1.22	9.11 ± 1.18	10.19 ± 0.65	10.45 ± 2.02	15.52 ± 1.82 *	22.59 ± 2.72 **
Peppermint	7.52 ± 1.81	8.76 ± 0.84	8.12 ± 0.88	9.01 ± 1.05	8.10 ± 0.75	9.23 ± 0.44	12.26 ± 1.62 *	22.96 ± 2.68 **
Frankincense	8.82 ± 1.21	10.16 ± 1.94	9.36 ± 1.27	10.76 ± 1.83	15.46 ± 1.13 *	ND	ND	ND
PC	52.52 ± 2.83							
(**B**)								
Concentrations of EOs and EO blends (µg/mL)								
	0	8 × 10^−3^	1.7 × 10^−2^	3.3 × 10^−2^	6.5 × 10^−2^	0.13	0.26	0.52
OnGuard	8.09 ± 0.95	10.02 ± 2.18	9.22 ± 1.93	9.58 ± 1.55	10.02 ± 2.65	12.24 ± 2.01 *	17.82 ± 1.23 **	ND
Lavender	8.95 ± 0.71	8.28 ± 1.55	7.15 ± 0.77	8.85 ± 1.33	9.04 ± 1.03	11.32 ± 2.05	15.57 ± 0.70 ***	ND
Oregano	6.75 ± 0.63	7.02 ± 0.95	6.85 ± 1.23	8.79 ± 2.91	15.75 ± 0.93 *	20.00 ± 2.52 ***	ND	ND
Tea tree	7.52 ± 1.81	7.67 ± 0.91	8.09 ± 0.73	8.98 ± 1.52	8.10 ± 1.05	9.52 ± 0.64	12.85 ± 0.87 *	19.23 ± 1.19 **
Zengest	9.02 ± 1.25	7.98 ± 0.88	9.48 ± 1.72	10.28 ± 1.71	11.34 ± 3.27	30.09 ± 3.73 ***	ND	ND
Lemon	8.74 ± 0.78	9.86 ± 1.32	9.42 ± 1.20	10.01 ± 1.21	9.45 ± 1.21	11.07 ± 3.06	19.02 ± 2.11 **	27.89 ± 3.53 ***
Peppermint	9.03 ± 1.05	8.66 ± 0.72	8.56 ± 1.13	9.01 ± 1.11	8.98 ± 1.08	10.14 ± 1.51	17.78 ± 2.34 **	22.01 ± 1.30 **
Frankincense	10.23 ± 2.12	9.98 ± 1.53	10.06 ± 1.70	12.17 ± 2.80	20.48 ± 3.06 ***	ND	ND	ND
PC	52.52 ± 2.83							

ND: not detectable; PC: positive control—hydrogen peroxide (300 µmol/L).

**Table 3:** Minimum inhibitory concentrations (MIC; µg/mL) of EOs against tested LAB bacteria and *W. coagulans*.

Strains	OR	TT	OG
*L. plantarum*	0.05	0.5	0.5
*L. rhamnosus*	0.05	0.5	0.5
*L. pentosus*	0.05	0.25	0.25
*L. paracasei*	0.05	0.25	0.25
*L. brevis*	0.05	0.5	0.5
*W. coagulans*	0.05	0.5	0.5

OR: oregano; TT: tea tree; OG: OnGuard.

**Table 4:** Effect of EOs and EO blends at a concentration of 1.25 µg/mL on the survival of LAB and *W. coagulans*.

Strains (Log CFU/g)	Control	LE	P	L	F	ZG
*L. plantarum*	6.60 ± 0.05	7.61 ± 0.51	7.52 ± 0.55	7.60 ± 0.51	6.55 ± 0.05	6.61 ± 0.05
*L. rhamnosus*	6.22 ± 0.06	7.21 ± 0.52	7.22 ± 0.59	7.20 ± 0.51	6.22 ± 0.09	6.12 ± 0.05
*L. pentosus*	6.52 ± 0.09	6.12 ± 0.11	6.22 ± 0.12	6.42 ± 0.09	6.45 ± 0.11	6.35 ± 0.12
*L. paracasei*	6.85 ± 0.15	6.75 ± 0.14	6.65 ± 0.11	6.81 ± 0.11	6.75 ± 0.13	6.85 ± 0.11
*L. brevis*	6.74 ± 0.08	6.64 ± 0.11	6.71 ± 0.09	6.70 ± 0.12	6.68 ± 0.08	6.70 ± 0.08
*W. coagulans*	5.61 ± 0.11	5.66 ± 0.16	5.65 ± 0.12	5.56 ± 0.15	5.55 ± 0.12	5.62 ± 0.11

Notes: bacterial levels were determined by plate counts on De Man–Rogosa–Sharpe or nutrient agar plates; LE: lemon, P: peppermint, L: lavender, F: frankincense, ZG: EO blend Zengest. The results are presented as means ± SD of three independent experiments.

## Data Availability

Data are contained within this article.

## Supplementary Materials

The following supporting information can be downloaded at: https://www.mdpi.com/article/10.3390/molecules30040890/s1.
