# Supplementary material for: Evaluation of Bioactivity of Essential Oils: Cytotoxic/Genotoxic Effects on Colorectal Cancer Cell Lines, Antibacterial Activity, and Survival of Lactic Acid Bacteria
Source: Molecules. 2025 Feb 14;30(4):890. doi: 10.3390/molecules30040890 (PMC11858314; doi:10.3390/molecules30040890)
Supplement: Supplementary file 1 [file molecules-30-00890-s001.zip › Figure S1 Frankincense.pdf]

Aromatic Plant Research Center

We provide uncompromising
quality control for your products.

Customer : doTERRA International
Lot Number : 240881Y
Date Filled : 03/28/2024

Column : ZB5 (60 m length × 0.25 mm inner diameter × 0.25 μm film thickness)
Instrument : Shimadzu GCMS-QP2010 Ultra
Carrier gas : Helium 80 psi
Temperature ramp : 2 degrees celsius per minute up to 260-degrees celsius
Split ratio : 30:1
Sample preparation : 5%w/v solution with Dichloromethane.

Comments:
The analysis of this Frankincense lot revealed no contaminants or adulteration.
The sample meets the expected chemical profile for authentic essential oils of Boswellia species.

Analysied by : Ambika Poudel
Reviewed by : Dr. Prabodh Satyal

CERTIFIED BY
AROMATIC PLANT RESEARCH CENTER

© Copyright 2024 Aromatic Plant Research Center. All rights reserved. Any publishing, copying, use, dissemination, or distribution of this report, including online, without the express written permission of Aromatic Plant Research Center is strictly prohibited.

Aromatic Plant Research Center
230 N 1200 E STE 100
Lehi, UT 84043
www.aromaticplant.org

APRC®
Aromatic Plant Research Center

Frankincense Essential Oil

Customer : doTERRA International

Lot Number : 240881Y

Date Filled : 03/28/2024

Chromatogram

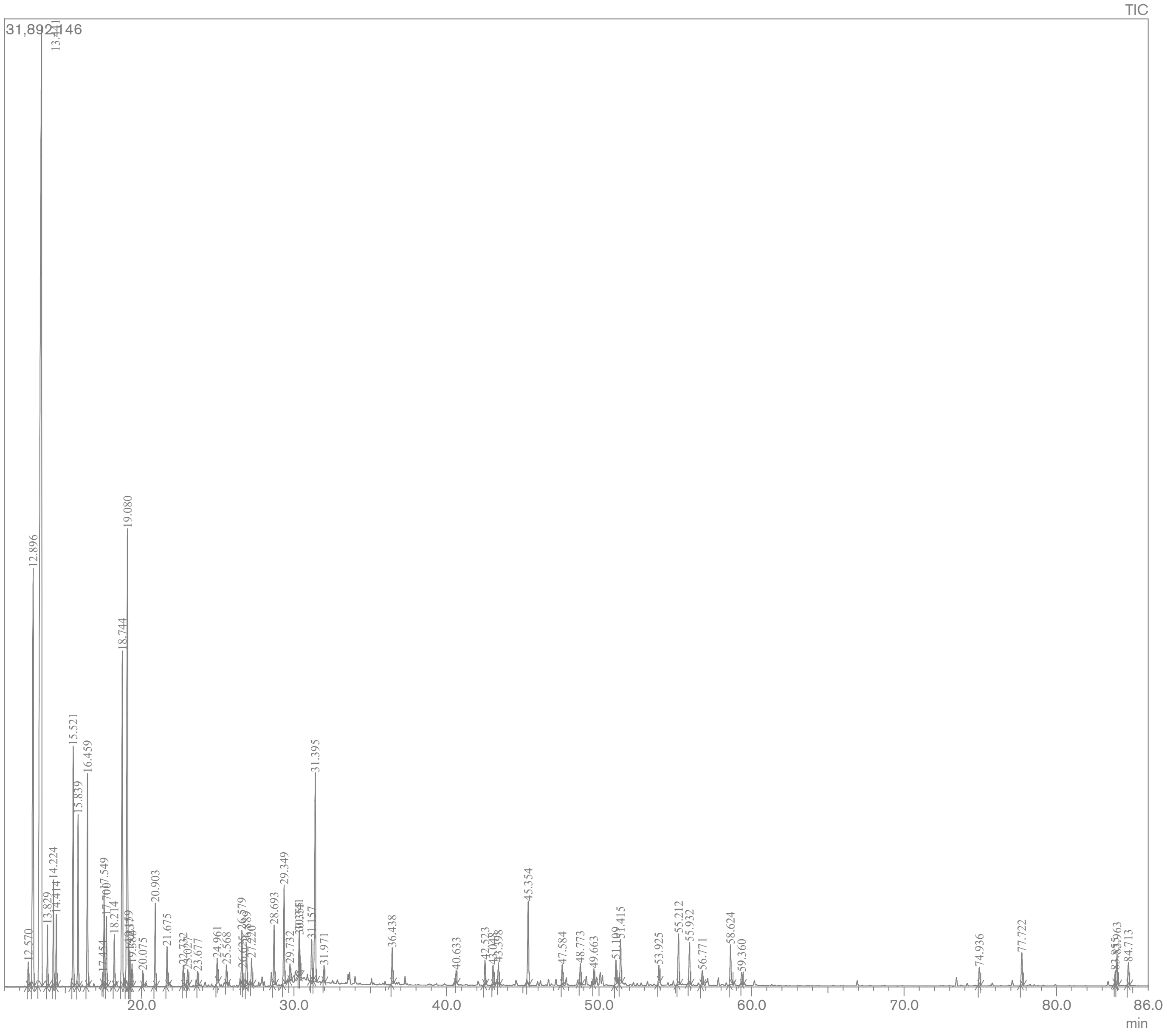

CERTIFIED BY AROMATIC PLANT RESEARCH CENTER

© Copyright 2024 Aromatic Plant Research Center. All rights reserved. Any publishing, copying, use, dissemination, or distribution of this report, including online, without the express written permission of Aromatic Plant Research Center is strictly prohibited.

Aromatic Plant Research Center
230 N 1200 E STE 100
Lehi, UT 84043
www.aromaticplant.org

APRC® Aromatic Plant Research Center

Peak Report

Peak#	R.Time	Name	Area%
1	12.570	Hashishene	0.34
2	12.896	alpha-Thujene	8.40
3	13.441	alpha-Pinene	33.24
4	13.829	Thujadiene	0.77
5	14.224	Camphene	1.38
6	14.414	Thuja-2,4(10)-diene	0.94
7	15.521	Sabinene	3.56
8	15.839	beta-Pinene	2.42
9	16.459	Myrcene	3.16
10	17.454	3-Ethenyl-1,2-dimethyl-1,4-cyclohexadiene	0.15
11	17.549	alpha-Phellandrene	1.37
12	17.700	delta-3-Carene	0.99
13	18.214	alpha-Terpinene	0.74
14	18.744	para-Cymene	6.13
15	19.080	Limonene	9.15
16	19.159	beta-Phellandrene	0.49
17	19.237	1,8-Cineole	0.45
18	19.380	cis-beta-Ocimene	0.35
19	20.075	trans-beta-Ocimene	0.21
20	20.903	gamma-Terpinene	1.27
21	21.675	n-Octanol	0.61
22	22.732	Terpinolene	0.33
23	23.027	para-Cymenene	0.25
24	23.677	Linalool	0.21
25	24.961	alpha-Thujone	0.41
26	25.568	alpha-Campholenal	0.26
27	26.579	trans-Pinocarveol	0.87
28	26.625	cis-Verbenol	0.13
29	26.889	trans-Verbenol	0.68
30	27.220	alpha-Felandren-8-ol	0.40
31	28.693	para-Mentha-1,5-dien-8-ol	1.04
32	29.349	Terpinen-4-ol	1.71
33	29.732	para-Cymen-8-ol	0.33
34	30.341	alpha-Terpineol	0.54
35	30.355	Myrtenol	0.46
36	31.157	Verbenone	0.69
37	31.395	Octyl acetate	4.05
38	31.971	trans-Carveol	0.23
39	36.438	Bornyl acetate	0.61
40	40.633	alpha-Cubebene	0.17
41	42.523	alpha-Copaene	0.44
42	43.048	beta-Bourbonene	0.36
43	43.398	beta-Elemene	0.34
44	45.354	beta-Caryophyllene	1.54
45	47.584	alpha-Humulene	0.36
46	48.773	trans-Cadina-1(6),4-diene	0.34
47	49.663	beta-Selinene	0.25
48	51.109	gamma-Cadinene	0.43
49	51.415	delta-Cadinene	0.80
50	53.925	trans-Nerolidol	0.30
51	55.212	Caryophyllene oxide	0.97
52	55.932	Viridiflorol	0.76
53	56.771	1,10-di-epi-Cubenol	0.27
54	58.624	epi-alpha-Cadinol	0.85
55	59.360	alpha-Cadinol	0.23
56	74.936	3-cis-Cembrene A	0.34
57	77.722	Verticilla-4(20),7,11-triene	0.66
58	83.855	Incensole	0.25
59	83.963	Serratol	0.61
60	84.713	Incensyl acetate	0.44
			100.00

CERTIFIED BY AROMATIC PLANT RESEARCH CENTER

© Copyright 2024 Aromatic Plant Research Center. All rights reserved. Any publishing, copying, use, dissemination, or distribution of this report, including online, without the express written permission of Aromatic Plant Research Center is strictly prohibited.
