# Supplementary material for: Evaluation of Bioactivity of Essential Oils: Cytotoxic/Genotoxic Effects on Colorectal Cancer Cell Lines, Antibacterial Activity, and Survival of Lactic Acid Bacteria
Source: Molecules. 2025 Feb 14;30(4):890. doi: 10.3390/molecules30040890 (PMC11858314; doi:10.3390/molecules30040890)
Supplement: Supplementary file 1 [file molecules-30-00890-s001.zip › Figure S2 Lavender.pdf]

Aromatic Plant Research Center

We provide uncompromising
quality control for your products.

Customer : doTERRA International
Lot Number : 240961Y
Date Filled : 04/05/2024

Column : ZB5 (60 m length × 0.25 mm inner diameter × 0.25 µm film thickness)
Instrument : Shimadzu GCMS-QP2010 Ultra
Carrier gas : Helium 80 psi
Temperature ramp : 2 degrees celsius per minute up to 260-degrees celsius
Split ratio : 30:1
Sample preparation : 5%w/v solution with Dichloromethane.

Comments:
The analysis of this Lavender lot revealed no contaminants or adulteration.
The sample meets the expected chemical profile for authentic essential oils of Lavandula angustifolia.

Analysied by: Ambika Poudel
Reviewed by: Dr. Prabodh Satyal

© Copyright 2024 Aromatic Plant Research Center. All rights reserved. Any publishing, copying, use, dissemination, or distribution of this report including online, without the express written permission of Aromatic Plant Research Center is strictly prohibited.

Aromatic Plant Research Center
230 N 1200 E STE 100
Lehi, UT 84043
www.aromaticplant.org

APRC®
Aromatic Plant Research Center

Lavender Essential Oil

Customer : doTERRA International

Lot Number : 240961Y

Date Filled : 04/05/2024

Chromatogram

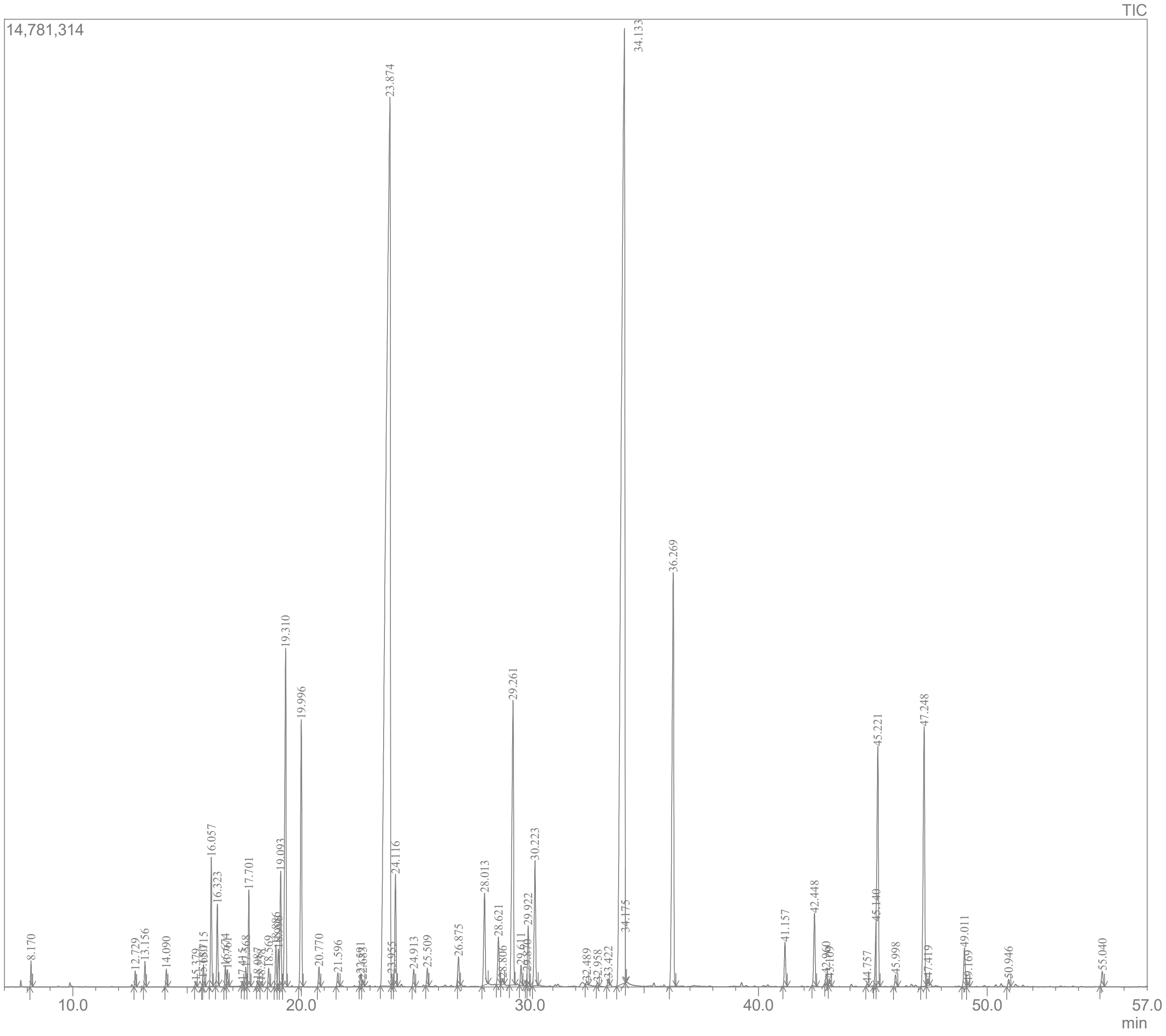

CERTIFIED BY
AROMATIC PLANT RESEARCH CENTER

© Copyright 2024 Aromatic Plant Research Center. All rights reserved. Any publishing, copying, use, dissemination, or distribution of th
including online, without the express written permission of Aromatic Plant Research Center is strictly prohibited.

Aromatic Plant Research Center
230 N 1200 E STE 100
Lehi, UT 84043
www.aromaticplant.org

APRC®
Aromatic Plant Research Center

Peak Report

Peak#	R.Time	Name	Area%
1	8.170	1-Methoxy hexane	0.16
2	12.729	alpha-Thujene	0.13
3	13.156	alpha-Pinene	0.21
4	14.090	Camphene	0.15
5	15.379	Sabinene	0.04
6	15.680	beta-Pinene	0.02
7	15.715	1-Octen-3-ol	0.20
8	16.057	3-Octanone	1.22
9	16.323	Myrcene	0.72
10	16.674	Butyl butanoate	0.18
11	16.761	3-Octanol	0.15
12	17.415	alpha-Phellandrene	0.04
13	17.568	delta-3-Carene	0.17
14	17.701	Hexyl acetate	0.89
15	18.087	alpha-Terpinene	0.05
16	18.238	meta-Cymene	0.04
17	18.569	para-Cymene	0.17
18	18.886	Limonene	0.42
19	18.996	beta-Phellandrene	0.36
20	19.093	1,8-Cineole	1.11
21	19.310	cis-beta-Ocimene	3.86
22	19.996	trans-beta-Ocimene	2.85
23	20.770	gamma-Terpinene	0.19
24	21.596	cis-Linalool oxide (furanoid)	0.16
25	22.591	Terpinolene	0.12
26	22.683	trans-Linalool oxide (furanoid)	0.08
27	23.874	Linalool	32.39
28	23.955	Hexyl propionate+Hotrienol	0.11
29	24.116	1-Octen-3-yl acetate	1.12
30	24.913	3-Octanol acetate	0.18
31	25.509	allo-Ocimene	0.18
32	26.875	Camphor	0.31
33	28.013	Lavandulol	1.13
34	28.621	Borneol	0.58
35	28.806	3-trans-5-cis-1,3,5-Undecatriene	0.08
36	29.261	Terpinen-4-ol	4.11
37	29.611	Cryptone	0.22
38	29.870	Butyl n-hexanoate	0.05
39	29.922	Hexyl butanoate	0.64
40	30.223	alpha-Terpineol	1.51
41	32.489	Bornyl formate	0.04
42	32.958	Hexyl 2-methyl butanoate	0.04
43	33.422	Cuminal	0.07
44	34.133	Linalyl acetate	27.97
45	34.175	Geraniol	0.07
46	36.269	Lavandulyl acetate	6.03
47	41.157	Neryl acetate	0.49
48	42.448	Geranyl acetate	0.82
49	42.960	Hexyl hexanoate	0.11
50	43.109	7-epi-Sesquithujene	0.07
51	44.757	cis-alpha-Bergamotene	0.03
52	45.140	alpha-Santalene	0.41
53	45.221	beta-Caryophyllene	3.16
54	45.998	trans-alpha-Bergamotene	0.14
55	47.248	trans-beta-Farnesene	3.38
56	47.419	alpha-Humulene	0.08
57	49.011	Germacrene D	0.47
58	49.169	trans-beta-Bergamotene	0.04
59	50.946	gamma-Cadinene	0.09
60	55.040	Caryophyllene oxide	0.20
			100.00

CERTIFIED BY AROMATIC PLANT RESEARCH CENTER

© Copyright 2024 Aromatic Plant Research Center. All rights reserved. Any publishing, copying, use, dissemination, or distribution of th
including online, without the express written permission of Aromatic Plant Research Center is strictly prohibited.
