# Supplementary material for: Evaluation of Bioactivity of Essential Oils: Cytotoxic/Genotoxic Effects on Colorectal Cancer Cell Lines, Antibacterial Activity, and Survival of Lactic Acid Bacteria
Source: Molecules. 2025 Feb 14;30(4):890. doi: 10.3390/molecules30040890 (PMC11858314; doi:10.3390/molecules30040890)
Supplement: Supplementary file 1 [file molecules-30-00890-s001.zip › Figure S3 Lemon.pdf]

Aromatic Plant Research Center

We provide uncompromising
quality control for your products.

Customer : doTERRA International
Lot Number : 240091
Date Filled : 01/09/2024

Column : ZB5 (60 m length × 0.25 mm inner diameter × 0.25 μm film thickness)
Instrument : Shimadzu GCMS-QP2010 Ultra
Carrier gas : Helium 80 psi
Temperature ramp : 2 degrees celsius per minute up to 260-degrees celsius
Split ratio : 30:1
Sample preparation : 5%w/v solution with Dichloromethane.

Comments:
The analysis of this Lemon lot revealed no contaminants or adulteration.
The sample meets the expected chemical profile for authentic essential oils of Citrus limon.

Analysied by : Ambika Poudel
Reviewed by : Dr. Prabodh Satyal

CERTIFIED BY AROMATIC PLANT RESEARCH CENTER

© Copyright 2024 Aromatic Plant Research Center. All rights reserved. Any publishing, copying, use, dissemination, or distribution of this report, including online, without the express written permission of Aromatic Plant Research Center is strictly prohibited.

Aromatic Plant Research Center
230 N 1200 E STE 100
Lehi, UT 84043
www.aromaticplant.org

Lemon Essential Oil

Customer : doTERRA International

Lot Number : 240091

Date Filled : 01/09/2024

Chromatogram

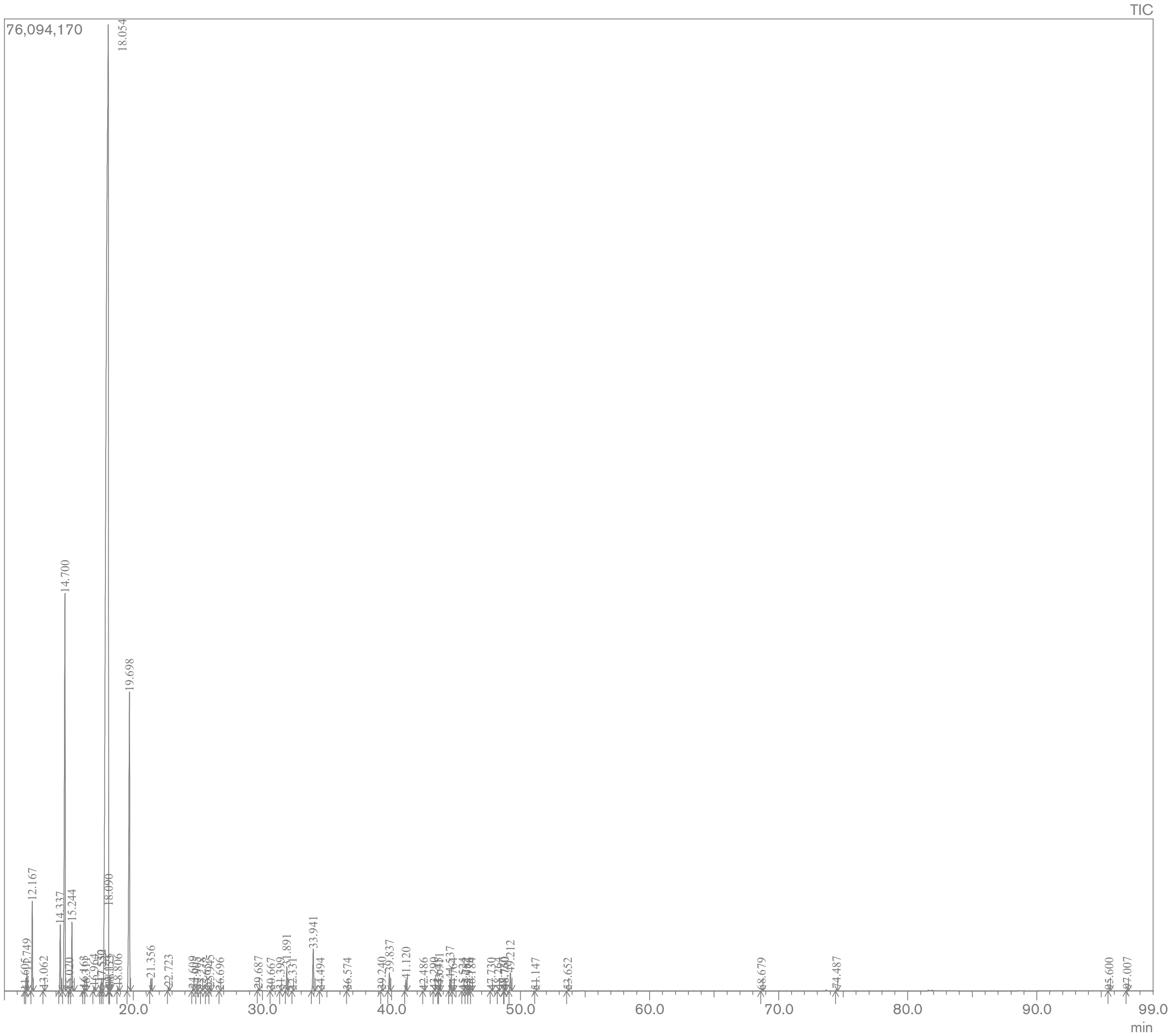

© Copyright 2024 Aromatic Plant Research Center. All rights reserved. Any publishing, copying, use, dissemination, or distribution of this report, including online, without the express written permission of Aromatic Plant Research Center is strictly prohibited.

Aromatic Plant Research Center
230 N 1200 E STE 100
Lehi, UT 84043
www.aromaticplant.org

Peak Report

Peak#	R.Time	Name	Area%
1	11.605	Tricyclene	0.00
2	11.749	alpha-Thujene	0.33
3	12.167	alpha-Pinene	1.50
4	13.062	Camphene	0.03
5	14.337	Sabinene	1.41
6	14.700	beta-Pinene	9.99
7	15.020	6-Methyl-5-hepten-2-one	0.01
8	15.244	Myrcene	1.31
9	16.163	n-Octanal	0.05
10	16.311	alpha-Phellandrene	0.02
11	16.964	alpha-Terpinene	0.11
12	17.530	meta-Cymene	0.22
13	17.552	para-Cymene	0.28
14	18.054	Limonene	71.95
15	18.090	beta-Phellandrene	0.29
16	18.125	1,8-Cineole	0.03
17	18.153	cis-beta-Ocimene	0.03
18	18.806	trans-beta-Ocimene	0.09
19	19.698	gamma-Terpinene	8.31
20	21.356	Terpinolene	0.25
21	22.723	n-Nonanal	0.07
22	24.609	cis-Limonene oxide	0.04
23	24.905	trans-Limonene oxide	0.03
24	25.278	Epoxy terpinolene	0.02
25	25.657	Camphor	0.00
26	25.945	Citronellal	0.07
27	26.696	trans-trans-2,6-Dimethyl-3,5,7-octatriene-2-ol	0.01
28	29.687	n-Decanal	0.03
29	30.667	Epoxy neral	0.01
30	31.399	Epoxy geranial	0.02
31	31.891	Neral	0.56
32	32.331	Carvone	0.01
33	33.941	Geranial	1.05
34	34.494	Perilla aldehyde	0.01
35	36.574	Undecanal	0.01
36	39.240	Citronellyl acetate	0.03
37	39.837	Neryl acetate	0.43
38	41.120	Geranyl acetate	0.26
39	42.486	Tetradecane	0.01
40	43.290	cis-alpha-Bergamotene	0.02
41	43.645	alpha-Santalene	0.00
42	43.711	beta-Caryophyllene	0.13
43	44.537	trans-alpha-Bergamotene	0.29
44	44.764	Isocaryophyllene	0.01
45	45.524	Neryl propionate	0.00
46	45.782	trans-beta-Farnesene	0.02
47	45.976	alpha-Humulene	0.01
48	46.184	beta-Santalene	0.01
49	47.730	trans-beta-Bergamotene	0.01
50	48.264	Valencene	0.02
51	48.730	n-Pentadecane	0.01
52	48.760	cis-alpha-Bisabolene	0.03
53	49.212	beta-Bisabolene	0.44
54	51.147	trans-alpha-Bisabolene	0.01
55	53.652	Caryophyllene oxide	0.01
56	68.679	Pentadecanoic acid	0.01
57	74.487	Citropten	0.04
58	95.600	Oxypeucedanin	0.01
59	97.007	Isooxypeucedanine	0.03
			100.00

© Copyright 2024 Aromatic Plant Research Center. All rights reserved. Any publishing, copying, use, dissemination, or distribution of this report, including online, without the express written permission of Aromatic Plant Research Center is strictly prohibited.
