# Supplementary material for: Evaluation of Bioactivity of Essential Oils: Cytotoxic/Genotoxic Effects on Colorectal Cancer Cell Lines, Antibacterial Activity, and Survival of Lactic Acid Bacteria
Source: Molecules. 2025 Feb 14;30(4):890. doi: 10.3390/molecules30040890 (PMC11858314; doi:10.3390/molecules30040890)
Supplement: Supplementary file 1 [file molecules-30-00890-s001.zip › Figure S4 Melaleuca.pdf]

Aromatic Plant Research Center

We provide uncompromising
quality control for your products.

Customer : doTERRA International
Lot Number : 200099
Date Filled : 01/09/2020

Column : ZB5 (60 m length × 0.25 mm inner diameter × 0.25 μm film thickness)
Instrument : Shimadzu GCMS-QP2010 Ultra
Carrier gas : Helium 80 psi
Temperature ramp : 2 degrees celsius per minute up to 260-degrees celsius
Split ratio : 30:1
Sample preparation : 5%w/v solution with Dichloromethane.

Comments:
The analysis of this Melaleuca lot revealed no contaminants or adulteration.
The sample meets the expected chemical profile for authentic essential oils of Melaleuca alternifolia.

Analyzed by: Ambika Poudel
Reviewed by: Dr. Prabodh Satyal

CERTIFIED BY
AROMATIC PLANT RESEARCH CENTER

© Copyright 2020 Aromatic Plant Research Center. All rights reserved. Any publishing, copying, use, dissemination, or distribution of this report, including online, without the express written permission of Aromatic Plant Research Center is strictly prohibited.

Aromatic Plant Research Center
230 N 1200 E STE 100
Lehi, UT 84043
www.aromaticplant.org

APRC®
Aromatic Plant Research Center

Melaleuca Essential Oil

Customer : doTERRA International

Lot Number : 200099

Date Filled : 01/09/2020

Chromatogram

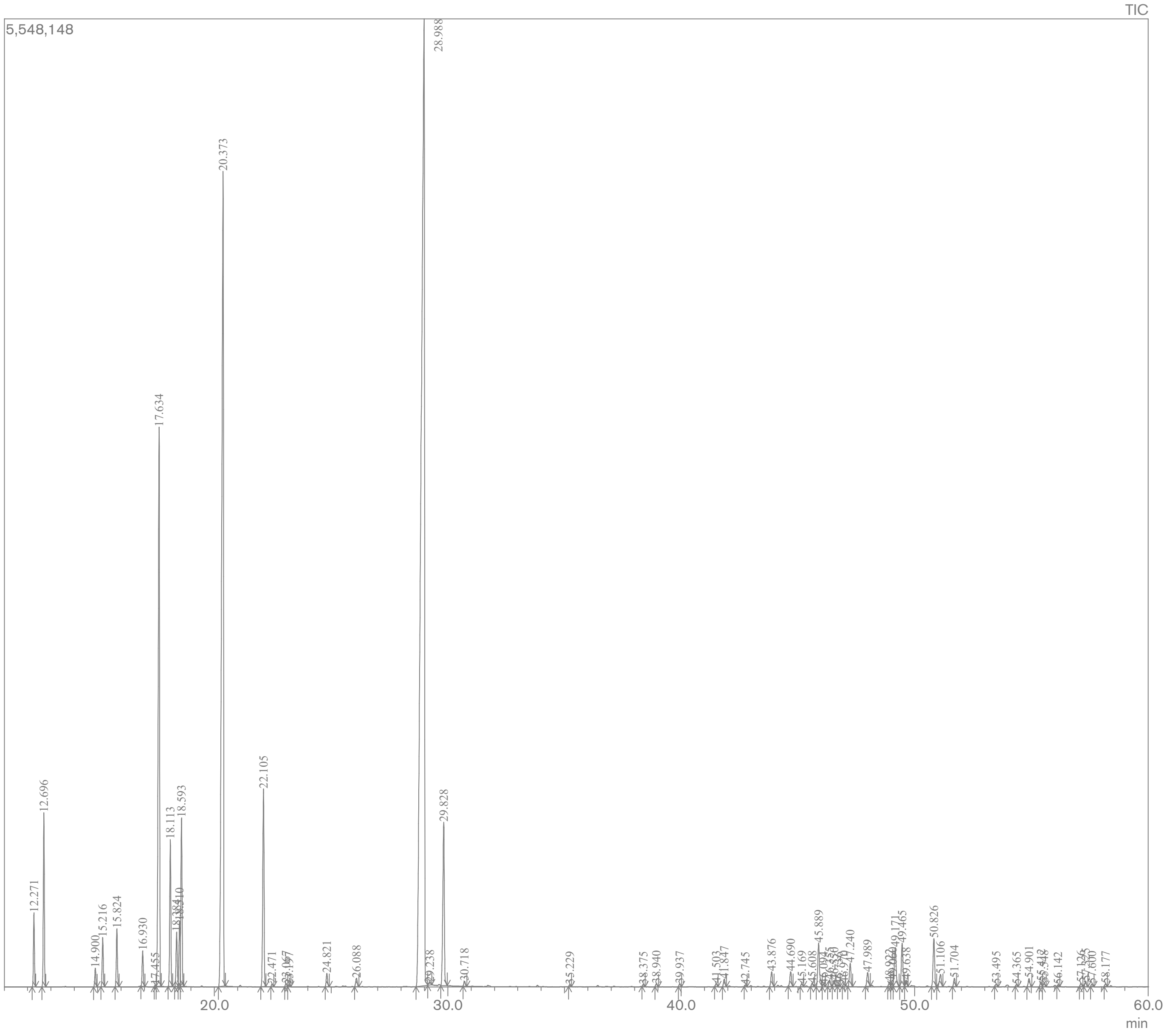

CERTIFIED BY AROMATIC PLANT RESEARCH CENTER

© Copyright 2020 Aromatic Plant Research Center. All rights reserved. Any publishing, copying, use, dissemination, or distribution of this report, including online, without the express written permission of Aromatic Plant Research Center is strictly prohibited.

Aromatic Plant Research Center
230 N 1200 E STE 100
Lehi, UT 84043
www.aromaticplant.org

Peak Report

Peak#	R.Time	Name	Area%
1	12.271	alpha-Thujene	0.97
2	12.696	alpha-Pinene	2.34
3	14.900	Sabinene	0.26
4	15.216	beta-Pinene	0.72
5	15.824	Myrcene	0.84
6	16.930	alpha-Phellandrene	0.54
7	17.455	1,4-Cineole	0.02
8	17.634	alpha-Terpinene	10.04
9	18.113	para-Cymene	2.21
10	18.384	Limonene	0.88
11	18.510	beta-Phellandrene	1.02
12	18.593	1,8-cineole	2.66
13	20.373	gamma-Terpinene	19.75
14	22.105	Terpinolene	3.37
15	22.471	para-Cymenene	0.05
16	23.067	Linalool	0.05
17	23.197	trans-Sabinene hydrate	0.03
18	24.821	cis-para-Menth-2-en-1-ol	0.24
19	26.088	trans-para-Menth-2-en-1ol	0.17
20	28.988	Terpinen-4-ol	42.77
21	29.238	para-Cymen-8-ol	0.06
22	29.828	alpha-Terpineol	3.24
23	30.718	trans-Piperitol	0.09
24	35.229	trans-Ascaridol glycol	0.03
25	38.375	4-isopropyl-6-methyl-3-cyclohexene-1,2-diol	0.04
26	38.940	Bicylcoelemene	0.05
27	39.937	alpha-Cubebene	0.05
28	41.503	Isoledene	0.06
29	41.847	alpha-Copaene	0.15
30	42.745	beta-Elemene	0.03
31	43.876	alpha-Gurjunene	0.30
32	44.690	beta-Caryophyllene	0.31
33	45.169	cis-9-epi-Caryophyllene	0.06
34	45.608	alpha-Maaliene	0.06
35	45.889	Aromadendrene	1.01
36	46.094	alpha-Guaiene	0.04
37	46.355	Selina5,11-diene	0.14
38	46.550	trans-Murrola-3,5-diene	0.12
39	46.736	Velerana7,11-diene	0.02
40	46.970	alpha-Humulene	0.05
41	47.240	Alloaromadendrene	0.47
42	47.989	cis-Cadina-1(6),4-diene	0.30
43	48.922	delta-Selinene	0.09
44	49.060	beta-Selinene	0.13
45	49.171	Viridiflorene	0.94
46	49.465	Bicyclogermacrene	0.93
47	49.638	alpha-Muurolene	0.12
48	50.826	delta-Cadinene	0.98
49	51.106	Zonarene	0.30
50	51.704	trans-Cadine-1,4-diene	0.16
51	53.495	Viridifloral isomer	0.05
52	54.365	Spathulenol	0.04
53	54.901	Globulol	0.18
54	55.412	Viridiflorol	0.10
55	55.548	Cubeban-11-ol	0.07
56	56.142	Rosifoliol	0.02
57	57.126	Eudesmol isomer	0.06
58	57.325	1-epi-Cubenol	0.11
59	57.600	iso-Spathulenol	0.05
60	58.177	Cubenol	0.05
			100.00

CERTIFIED BY AROMATIC PLANT RESEARCH CENTER

© Copyright 2020 Aromatic Plant Research Center. All rights reserved. Any publishing, copying, use, dissemination, or distribution of this report, including online, without the express written permission of Aromatic Plant Research Center is strictly prohibited.
