# Supplementary material for: Evaluation of Bioactivity of Essential Oils: Cytotoxic/Genotoxic Effects on Colorectal Cancer Cell Lines, Antibacterial Activity, and Survival of Lactic Acid Bacteria
Source: Molecules. 2025 Feb 14;30(4):890. doi: 10.3390/molecules30040890 (PMC11858314; doi:10.3390/molecules30040890)
Supplement: Supplementary file 1 [file molecules-30-00890-s001.zip › Figure S5 Oregano.pdf]

Aromatic Plant Research Center

We provide uncompromising
quality control for your products.

**Customer**	: doTERRA International
**Lot Number**	: 230612
**Date Filled**	: 03/02/2023

**Column**	: ZB5 (60 m length × 0.25 mm inner diameter × 0.25 µm film thickness)
**Instrument**	: Shimadzu GCMS-QP2010 Ultra
**Carrier gas**	: Helium 80 psi
**Temperature ramp**	: 2 degrees celsius per minute up to 260-degrees celsius
**Split ratio**	: 30:1
**Sample preparation**	: 5%w/v solution with Dichloromethane.

Comments:

The analysis of this Oregano lot revealed no contaminants or adulteration.
The sample meets the expected chemical profile for authentic essential oils of Origanum vulgare.

Analysied by : Ambika Poudel
Reviewed by : Dr. Prabodh Satyal

CERTIFIED BY AROMATIC PLANT RESEARCH CENTER

© Copyright 2023 Aromatic Plant Research Center. All rights reserved. Any publishing, copying, use, dissemination, or distribution of this report, including online, without the express written permission of Aromatic Plant Research Center is strictly prohibited.

Oregano Essential Oil

Customer : doTERRA International

Lot Number : 230612

Date Filled : 03/02/2023

Chromatogram

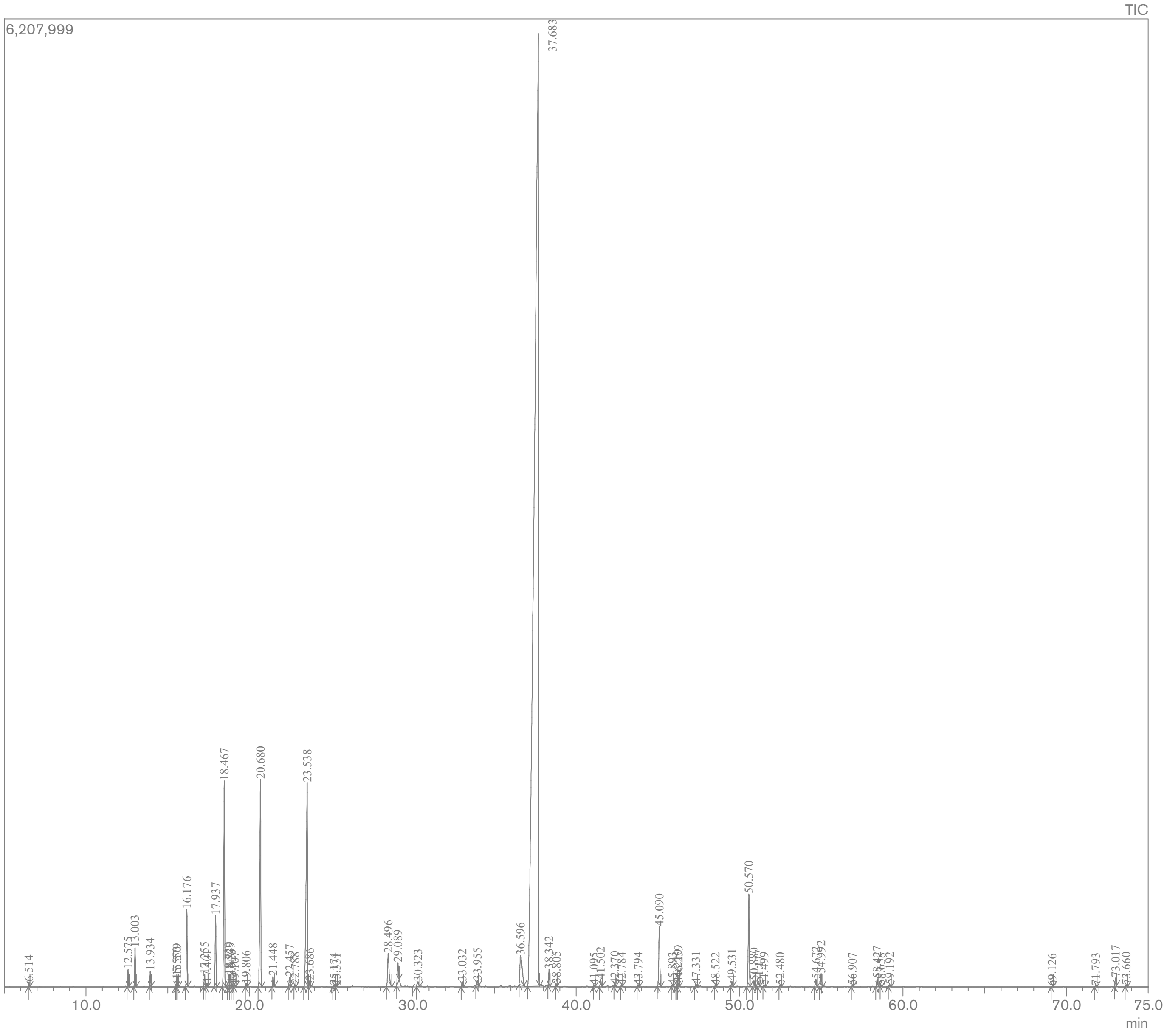

© Copyright 2023 Aromatic Plant Research Center. All rights reserved. Any publishing, copying, use, dissemination, or distribution of this report, including online, without the express written permission of Aromatic Plant Research Center is strictly prohibited.

Aromatic Plant Research Center
230 N 1200 E STE 100
Lehi, UT 84043
www.aromaticplant.org

APRC®
Aromatic Plant Research Center

Peak Report

Peak#	R.Time	Name	Area%
1	6.514	Methyl alpha-methyl butyrate	0.04
2	12.575	alpha-Thujene	0.22
3	13.003	alpha-Pinene	0.52
4	13.934	Camphene	0.21
5	15.550	beta-Pinene	0.06
6	15.579	1-Octen-3-ol	0.15
7	16.176	Myrcene	1.11
8	17.255	alpha-Phellandrene	0.18
9	17.401	delta-3-Carene	0.06
10	17.937	alpha-Terpinene	1.10
11	18.467	para-Cymene	3.57
12	18.729	Limonene	0.18
13	18.839	beta-Phellandrene	0.18
14	18.926	1,8-Cineole	0.06
15	19.107	cis-beta-Ocimene	0.03
16	19.806	trans-beta-Ocimene	0.05
17	20.680	gamma-Terpinene	3.88
18	21.448	cis-Sabinene hydrate	0.18
19	22.457	Terpinolene	0.16
20	22.788	para-Cymenene	0.03
21	23.538	Linalool	5.18
22	23.686	Hotrienol	0.13
23	25.174	cis-para-Menth-2-en-1-ol	0.02
24	25.331	alpha-Campholenal	0.02
25	28.496	Borneol	0.94
26	29.089	Terpinen-4-ol	0.41
27	30.323	alpha-Terpineol	0.12
28	33.032	Thymol methyl ether	0.09
29	33.955	Carvone	0.13
30	36.596	Thymol	1.25
31	37.683	Carvacrol	74.62
32	38.342	Methyl non-8-enoate	0.27
33	38.805	Oregano sesquiterpenoid 1	0.05
34	41.095	Neryl acetate	0.02
35	41.502	Thymol acetate	0.13
36	42.370	Geranyl acetate	0.04
37	42.784	beta-Bourbonene	0.03
38	43.794	Methyl eugenol	0.03
39	45.090	beta-Caryophyllene	1.17
40	45.893	trans-alpha-Bergamotene	0.03
41	46.210	Oregano sesquiterpenoid 2	0.05
42	46.259	Aromadendrene	0.16
43	47.331	alpha-Humulene	0.05
44	48.522	trans-Cadina-1(6),4-diene	0.02
45	49.531	Viridiflorene	0.09
46	50.570	beta-Bisabolene	1.83
47	50.880	gamma-Cadinene	0.12
48	51.177	delta-Cadinene	0.08
49	51.499	beta-Sesquiphellandrene	0.03
50	52.480	trans-alpha-Bisabolene	0.03
51	54.672	Spathulenol	0.15
52	54.992	Caryophyllene oxide	0.26
53	56.907	1,10-Di-epi-cubenol	0.02
54	58.427	epi-alpha-Cadinol	0.15
55	58.658	allo-Aromadendrene epoxide	0.02
56	59.192	alpha-Cadinol	0.04
57	69.126	Phytone	0.01
58	71.793	Oregano sesquiterpenoid 3	0.03
59	73.017	Oregano sesquiterpenoid 4	0.15
60	73.660	Oregano sesquiterpenoid 5	0.03
			100.00

CERTIFIED BY AROMATIC PLANT RESEARCH CENTER

© Copyright 2023 Aromatic Plant Research Center. All rights reserved. Any publishing, copying, use, dissemination, or distribution of this report, including online, without the express written permission of Aromatic Plant Research Center is strictly prohibited.
