# Supplementary material for: Evaluation of Bioactivity of Essential Oils: Cytotoxic/Genotoxic Effects on Colorectal Cancer Cell Lines, Antibacterial Activity, and Survival of Lactic Acid Bacteria
Source: Molecules. 2025 Feb 14;30(4):890. doi: 10.3390/molecules30040890 (PMC11858314; doi:10.3390/molecules30040890)
Supplement: Supplementary file 1 [file molecules-30-00890-s001.zip › Figure S6 Peppermint.pdf]

Aromatic Plant Research Center

We provide uncompromising
quality control for your products.

Customer : doTERRA International
Lot Number : 233171
Date Filled : 12/13/2023

Column : ZB5 (60 m length × 0.25 mm inner diameter × 0.25 µm film thickness)
Instrument : Shimadzu GCMS-QP2010 Ultra
Carrier gas : Helium 80 psi
Temperature ramp : 2 degrees celsius per minute up to 260-degrees celsius
Split ratio : 30:1
Sample preparation : 5%w/v solution with Dichloromethane.

Comments:
The analysis of this Peppermint lot revealed no contaminants or adulteration.
The sample meets the expected chemical profile for authentic essential oils of Mentha piperita.

Analysied by : Ambika Poudel
Reviewed by : Dr. Prabodh Satyal

© Copyright 2023 Aromatic Plant Research Center. All rights reserved. Any publishing, copying, use, dissemination, or distribution of this report, including online, without the express written permission of Aromatic Plant Research Center is strictly prohibited.

Aromatic Plant Research Center
230 N 1200 E STE 100
Lehi, UT 84043
www.aromaticplant.org

APRC
Aromatic Plant Research Center

Peppermint Essential Oil

Customer : doTERRA International

Lot Number : 233171

Date Filled : 12/13/2023

Chromatogram

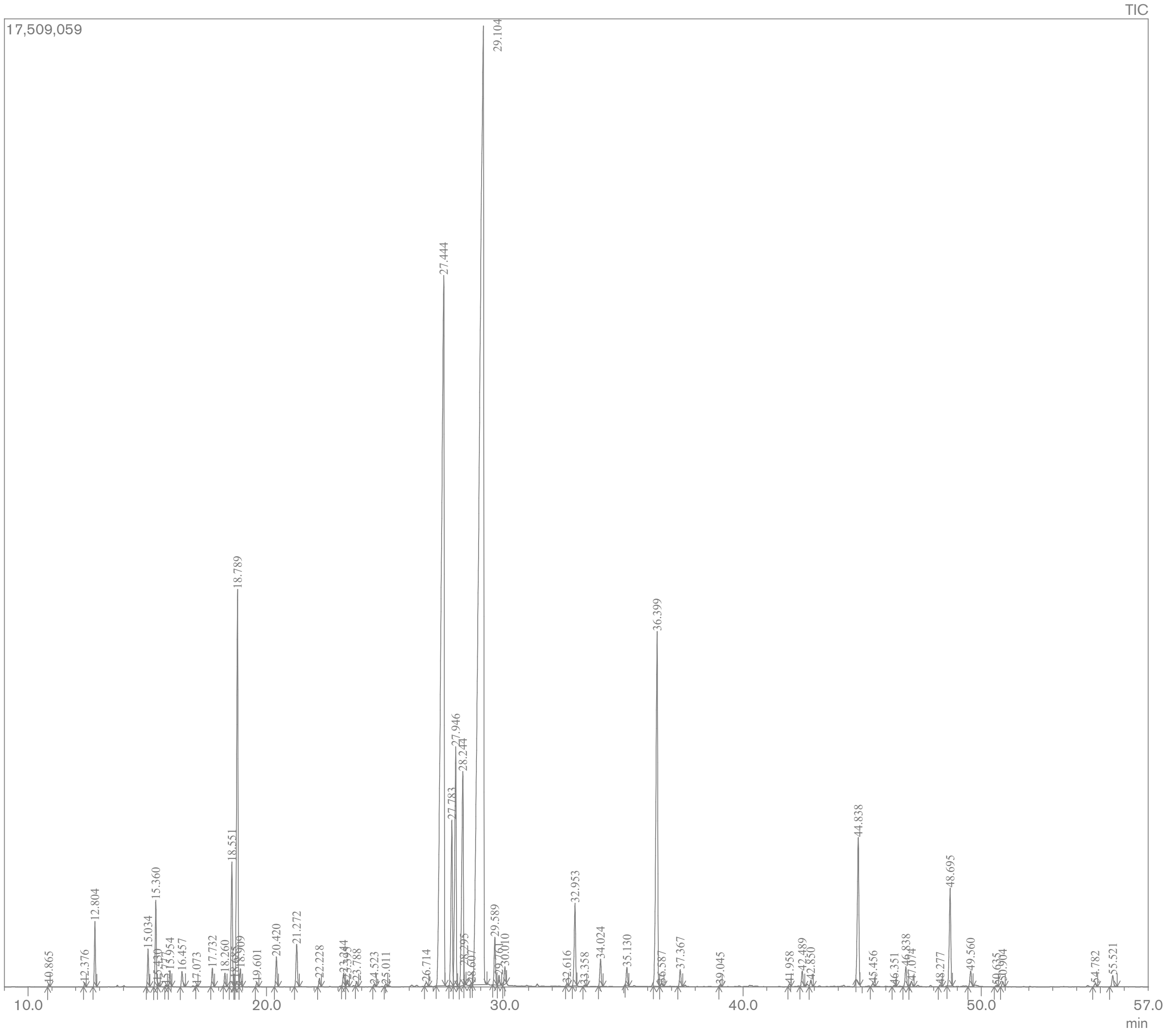

© Copyright 2023 Aromatic Plant Research Center. All rights reserved. Any publishing, copying, use, dissemination, or distribution of this report, including online, without the express written permission of Aromatic Plant Research Center is strictly prohibited.

Aromatic Plant Research Center
230 N 1200 E STE 100
Lehi, UT 84043
www.aromaticplant.org

APRC®
Aromatic Plant Research Center

Peak Report

Peak#	R.Time	Name	Area%
1	10.865	2,5-Diethyl tetrahydrofuran	0.03
2	12.376	alpha-Thujene	0.04
3	12.804	alpha-Pinene	0.66
4	15.034	Sabinene	0.41
5	15.360	beta-Pinene	0.97
6	15.430	1-Octen-3-ol	0.04
7	15.777	3-Octanone	0.02
8	15.954	Myrcene	0.18
9	16.457	3-Octanol	0.21
10	17.073	alpha-Phellandrene	0.01
11	17.732	alpha-Terpinene	0.22
12	18.260	para-Cymene	0.17
13	18.551	Limonene	1.71
14	18.685	beta-Phellandrene	0.04
15	18.789	1,8-Cineole	5.39
16	18.909	cis-beta-Ocimene	0.21
17	19.601	trans-beta-Ocimene	0.06
18	20.420	gamma-Terpinene	0.37
19	21.272	cis-Sabinene hydrate	0.60
20	22.228	Terpinolene	0.10
21	23.244	Linalool	0.21
22	23.395	2-Methyl butyl 2-methyl butyrate	0.10
23	23.788	2-Methyl butyl isovalerate	0.06
24	24.523	3-Octanol acetate	0.03
25	25.011	cis-para-Menth-2-en-1-ol	0.03
26	26.714	Isopulegol	0.07
27	27.444	Menthone	21.61
28	27.783	Menthofuran	2.36
29	27.946	Isomenthone	3.57
30	28.244	Neomenthol	3.56
31	28.295	delta-Terpineol	0.07
32	28.607	trans-Isopulegone	0.03
33	29.104	Menthol	42.46
34	29.589	iso-Menthol	0.61
35	29.761	neoiso-Menthol	0.13
36	30.010	alpha-Terpineol	0.23
37	32.616	3-cis-Hexenyl 3-methyl butanoate	0.03
38	32.953	Pulegone	1.16
39	33.358	Carvone	0.02
40	34.024	Piperitone	0.38
41	35.130	neo-Menthyl acetate	0.24
42	36.399	Menthyl acetate	6.20
43	36.587	Dihydroedulan	0.04
44	37.367	iso-Menthyl acetate	0.24
45	39.045	delta-Elemene	0.02
46	41.958	alpha-Copaene	0.04
47	42.489	beta-Bourbonene	0.23
48	42.850	beta-Elemene	0.09
49	44.838	beta-Caryophyllene	2.22
50	45.456	beta-Copaene	0.05
51	46.351	Aromadendrene	0.02
52	46.838	trans-beta-Farnesene	0.30
53	47.074	alpha-Humulene	0.08
54	48.277	trans-Cadina-1(6),4-diene	0.03
55	48.695	Germacrene D	1.47
56	49.560	Bicyclogermacrene	0.24
57	50.635	gamma-Cadinene	0.02
58	50.904	delta-Cadinene	0.08
59	54.782	Caryophyllene oxide	0.05
60	55.521	Viridiflorol	0.19
			100.00

CERTIFIED BY AROMATIC PLANT RESEARCH CENTER

© Copyright 2023 Aromatic Plant Research Center. All rights reserved. Any publishing, copying, use, dissemination, or distribution of this report, including online, without the express written permission of Aromatic Plant Research Center is strictly prohibited.
